# Mouse Hindbrain *Ex Vivo* Culture to Study Facial Branchiomotor Neuron Migration

**DOI:** 10.3791/51397

**Published:** 2014-03-18

**Authors:** Miguel Tillo, Quenten Schwarz, Christiana Ruhrberg

**Affiliations:** ^1^UCL Institute of Ophthalmology, University College London; ^2^Department of Human Immunology, Centre for Cancer Biology, South Australia

**Keywords:** Medicine, Issue 85, Neuroscience, Neuronal migration, hindbrain, mouse, facial branchiomotor neuron, vascular endothelial growth factor (VEGF)

## Abstract

Embryonic neurons are born in the ventricular zone of the brain, but subsequently migrate to new destinations to reach appropriate targets. Deciphering the molecular signals that cooperatively guide neuronal migration in the embryonic brain is therefore important to understand how the complex neural networks form which later support postnatal life. Facial branchiomotor (FBM) neurons in the mouse embryo hindbrain migrate from rhombomere (r) 4 caudally to form the paired facial nuclei in the r6-derived region of the hindbrain. Here we provide a detailed protocol for wholemount *ex vivo* culture of mouse embryo hindbrains suitable to investigate the signaling pathways that regulate FBM migration. In this method, hindbrains of E11.5 mouse embryos are dissected and cultured in an open book preparation on cell culture inserts for 24 hr. During this time, FBM neurons migrate caudally towards r6 and can be exposed to function-blocking antibodies and small molecules in the culture media or heparin beads loaded with recombinant proteins to examine roles for signaling pathways implicated in guiding neuronal migration.

**Figure:**
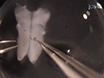


## Introduction

Embryonic neurons are born in the ventricular zone of the brain, but subsequently migrate to new destinations to reach appropriate target regions that are located at a large distance. The correct positioning of neuronal cell bodies in appropriate places along the dorso-ventral and anterior-posterior axes of the developing brain is essential for the correct wiring, survival, and function of these neurons after the migratory stage^1-4^. Similar to the molecular mechanisms that control axon guidance^5-7^, combinatorial sets of attractive and repulsive cues are thought to guide migrating neurons^1,8^. However, due to the interactions of multiple cell types, the signals controlling neuronal migration have been less extensively studied than those involved in axon guidance, which can be studied cell autonomously. The developing hindbrain of vertebrates has been used in several recent studies to understand the molecular and cellular mechanisms of neuronal migration, for example in chick, mouse, and zebrafish^1-4,9^. This organ contains several different types of neurons, including several subtypes of precerebellar and motor neurons^5,7,10,11^.

Hindbrain motorneuron are born in the ventricular zone close to the floorplate, and they differentiate into specific subsets according to their rhombomere of origin^1,12^. The facial branchiomotor (FBM) neurons are generated in rhombomere (r) 4 in the hindbrain and extend their axons dorsally through an r4 exit point into the second branchial arch to innervate the facial muscles^2,9,13^. FBM neurons of zebrafish and mice provide excellent models to study the molecular and cellular mechanisms of neuronal migration in a process that is readily visualized, because these neurons reproducibly translocate their somata in a spatiotemporally well-defined process. In mice, FBM neurons first migrate caudally through r5 and then both caudally and ventrally to reach their final position on the pial side of the hindbrain in the territory of r6, where they form the paired nuclei of the VIIth cranial nerve (VIIn)^10,11,14^. In zebrafish, FBM neurons initially migrate ventrally and then change direction at the r4-r5 boundary to continue migrating towards the pial surface in a laminin-dependent manner^4,12,15,16^. This migration proceeds over a period of several days in development and can be divided into phases of tangential and radial migration, allowing the identification of molecules that mediate these two distinct processes. In contrast, the FBM neurons of the embryonic chick hindbrain remain in r4^3,13,17-19^.

During their migration, FBM neurons can be identified, like other types or motor neurons, through their expression of the homoeodomain transcription factor islet 1 (ISL1)^14^. Thus, wholemount immunofluorescence staining or *in situ* hybridization for this marker at different developmental stages reveals the distinct migratory stream of FBM somata extending from r4 to r6 in the zebrafish or mouse^4,15,16^. Moreover, fluorescent transgenic reporters such as ISL1-GFP have been used as suitable tools to visualize migrating FBM neurons in zebrafish^3,17-19^. In addition to their suitability for imaging, many investigators have studied the migration of FBM neurons in developing zebrafish, because their free-living embryos can be manipulated easily with cell transplantation techniques and pharmacological compounds applied directly to the aquarium water. In contrast, the mouse embryo develops enclosed in the uterus, precluding the implantation of beads carrying guidance cues or the administration of function-blocking antibodies that do not cross the placental barrier. Moreover, pharmacological compounds administered to the pregnant mother may have undesired side effects that can indirectly impair embryogenesis. Circumventing this limitation, we have developed an *ex vivo* culture method for whole mouse hindbrain that is compatible with FBM neuron migration and survival for 24 hr after explanting^7,16^. This method allows easy pharmacological manipulation, implantation of beads carrying guidance cues or administration of function-blocking antibodies and could also be adapted to study the migration of other neuronal subtypes in the hindbrain at different developmental stages.

## Protocol

### 1. Optional: Prepare Affi-gel Heparin Beads (Gel Beads) for FBM Attraction Assay

NOTE: Prepare gel beads at least 1 day before starting the explant procedure.

1. Wash 100 μl gel heparin bead suspension with sterile PBS for 20 min on a roller at room temperature (RT).
2. Pellet beads in a table top centrifuge for 5 min at 13,000 x g. Add sterile PBS and repeat the washing procedure 4x.
3. After the final wash, remove PBS and soak the beads in a small volume of a sterile solution containing the recombinant protein of choice, taking care to cover the beads with the solution. This protocol uses 100 ng/μl recombinant human VEGF165 in PBS to reproduce a previously published experiment^16^.
4. Incubate the gel heparin beads with the recombinant protein solution for a minimum of 12 hr and a maximum of 1 week on a roller at 4 °C.

### 2. Coating of Culture Inserts

Hindbrain explants are cultured on Corning culture inserts with an 8 μm pore size, or equivalent inserts. Culture inserts can be reused after completion of the protocol, provided they are washed with distilled water, sterilized with ethanol, and stored in 70% ethanol until needed. NOTE: The following steps should be carried out in a flow hood under sterile conditions.

1. Prepare explant culture media consisting of Neurobasal medium supplemented with B27 (20 μl/ml), glucose (6 mg/ml) and penicillin/streptomycin (5 μg/μl).
2. Wash culture inserts with sterile PBS for 5 min and dry for 5-10 min under the flow hood.
3. Place one culture insert into each individual well of a 12well plate. Note: inserts may need a small push to fit tightly into the well.
4. Cover the culture inserts with 10-20 μg/ml mouse laminin in Neurobasal medium and place them in a tissue culture incubator (37 °C, 5% CO_2_) for 1 hr. Note: Coating should be carried out on the day of explanting.

### 3. Dissection of Hindbrains from E11.5 Mouse Embryos

1. Cull timed pregnant female mouse with an ethically approved procedure on embryonic day (E) 11.5 and place the uterus containing the embryos in a 100 mm plastic dish with ice-cold L15 medium. NOTE: All dissection steps must be performed in ice-cold L15.
2. Using a dissecting microscope and Dumont watchmaker forceps number 5, tear the uterine muscle wall to expose the embryos, release each embryo, sever the umbilical cord, and carefully remove the yolk sac.
3. Using a plastic Pasteur pipette with a wide-bore opening, transfer each embryo into a clean plastic dish with ice-cold L15.
4. Using Dumont forceps, decapitate the embryo just above the forelimbs. If the experiment requires genotyping of the embryos, collect tissue samples for genomic DNA isolation (*e.g.* a small piece of yolk sac or tail tip^16,20^).
5. Turn the head dorsal side up and identify the 4^th^ ventricle, which is covered by a thin tissue layer (**Figure 2B**). Carefully pierce the roofplate and begin to peel it away caudally along the midline over the posterior hindbrain and spinal cord, and rostrally over the midbrain. The hindbrain should now be exposed (**Figure 2C**).
6. Carefully tease away the remaining head mesenchyme and any meninges that are attached to the pial side of the hindbrain (**Figure 2D**).
7. Remove midbrain and spinal cord tissue so that the hindbrain unfurls and can lie flat in an open book preparation (**Figure 2E**).
8. Using a wide-bore plastic Pasteur pipette, transfer each dissected hindbrain to a 12-well plate containing ice-cold L15 and store them on ice until all hindbrains have been dissected.
9. Using a wide-bore plastic Pasteur pipette, transfer a single hindbrain to an empty dish, keeping it an open book preparation, ventricular side up, in a droplet of approximate 100 μl L15.
10. Transfer a few microliters of the incubated gel heparin beads to the same droplet. Note: Because the sizes of beads are variable, it is advisable to transfer around 10 beads and then choose an optimal size for transplantation into the hindbrain.
11. Make a small tear in the hindbrain tissue and carefully insert 1-3 gel beads into the hindbrain tissue at the level of r5/6, about half way between the midline and the lateral edge of the hindbrain, lowering them into the tissue so that the bead is positioned just beneath the hindbrain surface. NOTE: The dissection procedure may take between 5-20 min/hindbrain, depending on experience, and may stretch over an extended period if litters are large; in any case, hindbrains should be in culture no longer than 3 hr post-mortem for good results.

### 4. Hindbrain Explant Culture

1. Remove the plate containing the culture inserts from the incubator, aspirate the laminin coating solution.
2. Place one culture insert into a separate culture dish filled with ice-cold L15 and, using a wide-bore plastic Pasteur pipette, transfer each hindbrain ventral side up onto the culture insert (**Figure 2G**). The hindbrain should lie completely flat on the insert membrane.
3. Carefully lift the culture insert from the dish and dab it several times on clean tissue paper to remove excess liquid. This process ensures that the hindbrain adheres to the culture insert in a flat, open book preparation. If the hindbrain curls up, its tissue may irreversibly grow together.
4. Fill the original 12-well plate with 500 μl prewarmed culture media and place the insert back into this well. Carefully adjust the volume with another 400-600 μl of media to just cover the hindbrain, ensuring that the hindbrain does not float off the membrane. If it floats, return to step 4.4 and repeat until the hindbrain remains attached to the membrane.
5. At this stage, it is possible to add biological inhibitors of interest to the media to study their effect on the migration of FBM neurons*. * NOTE: If implanting beads or administering other treatments, it is recommended to maintain at least 2 control explants under normal growth conditions per experiment to ensure that the experiment has been set up successfully.
6. Incubate explants for 24-30 hr in a tissue culture incubator (37 °C, 5% CO_2_).

### 5. Wholemount Immunofluorescent Staining of Hindbrain Explants

1. Aspirate the media from each well, rinse in PBS and fix for 2 hr at 4 °C with gentle agitation in ice-cold 4% formaldehyde (freshly prepared or freshly thawed 4% paraformaldehyde dissolved in PBS). Note: Do not try to remove hindbrain from the culture insert before fixation is complete.
2. Rinse 3x with PBS. Carefully peel the hindbrains from the culture inserts using Dumont forceps. Some explants are difficult to peel off, but can usually be lifted by applying gentle pressure through repeatedly expelling PBS from a pipette.
3. Transfer the hindbrains to 2.0 ml round-bottomed tubes for immunofluorescence labelling. Permeabilize hindbrains for 30 min at room temperature (RT) in PBS containing 0.1% TritonX-100 (PBT) with gentle rolling.
4. Incubate for 1 hr at RT in PBT containing 10% heat-inactivated normal goat serum with gentle rolling.
5. Incubate explants with gentle rolling at 4 °C for 5 days with primary antibody specific for ISL1, diluted 1:100 in PBT containing 1% heat-inactivated normal goat serum.
6. Wash the explants at RT 4x with PBT for 15 min each.
7. Incubate explants with gentle rolling at RT for 3 hr with fluorophore-conjugated goat anti-mouse antibody (*e.g.* Alexa Fluor 488 goat anti mouse, diluted 1:200) in PBT containing 1% heat-inactivated normal goat serum.
8. Wash the explants 4x at RT with PBT for 15 min each with gentle rolling.
9. Postfix the explants in 4% formaldehyde for 30 min at RT.
10. Cover a glass slide with 3 layers of black electrical tape and excise with a scalpel a small square of the layered tape to create a pocket for the explants; alternatively, use a depression glass slide.
11. Mount each hindbrain in SlowFade reagent into one pocket and cover with a glass coverslip, carefully avoiding to trap air bubbles and image using a laser scanning confocal microscope. NOTE: As an alternative to immunostaining, in situ hybridization with riboprobes that recognize FBMs (i.e. Isl1 or Phox2b) may be used to visualize FBM neurons^16,21^.

**Summary of steps and timing** Timed mating to obtain E11.25 pregnancies: ~14 days Optional: Bead preparation (Protocol 1): ~2 hr, on the day before embryo isolation Prepare culture inserts and media (Protocol 2): ~30 min, before embryo isolation Embryo isolation and hindbrain dissection (steps 3.1-3.4): ~10 min/embryo Hindbrain dissection (steps 3.5-3.7): ~5-10 min/hindbrain Explant procedure (steps 3.8-3.9): ~5-10 min/hindbrain Optional: bead implantation (steps 3.10-3.11): ~5-10 min/hindbrain Explant culture (step 4.7): 24 hr Fixation for antibody staining (step 5.1): 2 hr Staining procedure and imaging (Protocol 5): 5 days

## Representative Results

This section illustrates examples of results that can be obtained by studying FBM neuron migration in the mouse hindbrain through *ex vivo* culture. We show that the FBM neurons in explanted hindbrains from day 11 mouse embryos first undergo a tangential migration (**Figure 3A**) and then begin to assemble the facial motor nuclei (**Figure 3B**), similar to their behavior *in utero* (see **Figure 1**). We further demonstrate that the implantation of a VEGF165-soaked bead attracts FBM neurons (**Figures 3C **and** 3D**), as previously shown^16^. Importantly, this protocol allows studying FBM migration in the absence of blood vessels or vessel-derived factors that may influence FBM migration *in utero*, because the nonperfused vasculature degenerates in culture^16^. Thus, the unspecific blood cell labelling observed when using the ISL1 mouse antibody on freshly isolated mouse hindbrains (**Figure 1**) is no longer present in hindbrain tissue after 24 hr in culture (**Figures 3A-F**). Finally, we show two examples of hindbrains that were not explanted correctly and therefore contain FBM neurons in an abnormal distribution, either because the hindbrain was not explanted soon enough after embryo isolation (**Figure 3E**) or because the hindbrain tissue folded up in the transwell (**Figure 3F**). 


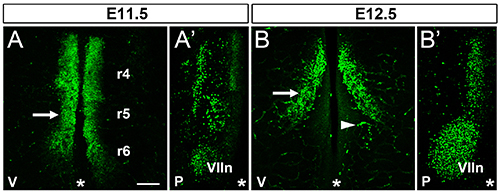
**Figure 1. FBM neuron migration.** Confocal z-stack of wildtype mouse hindbrains after ISL1 wholemount immunolabeling and flatmounting; the hindbrain midline is indicated with an asterisk in all panels. **(A)** Ventricular surface of an E11.5 hindbrain in the area containing ISL1-positive FBM neurons (arrow), demonstrating their tangential migration from r4 to r6; the position of r4, r5 and r6 is indicated. **(A’)** Pial surface of one half of the same hindbrain in the area containing the anlage of one of the paired FBM nuclei (indicated with VIIn), as well as other ISL1-positive neuron populations. **(B)** Ventricular surface of an E12.5 hindbrain containing FBM neurons that are migrating tangentially (arrow); the arrowhead indicate an example of a blood vessel containing circulating cells that are unspecifically labeled by cross-reaction of the anti-mouse secondary antibody used to detect the ISL1 mouse IgG antibody. **(B’)** Pial surface of one half of the same E12.5 hindbrain, which contains one of the paired FBM nuclei. The midline is indicated with an asterisk in each panel. Scale bar (all panels): 200 μm. V, ventral; P, pial. Click here to view larger image.


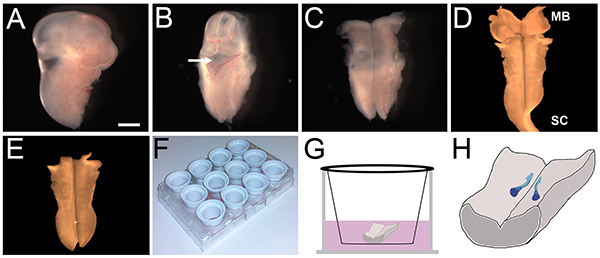
**Figure 2. E11.5 mouse hindbrain dissection and *ex vivo* culture. (A-E) **Key steps in the E11.5 hindbrain dissection protocol; scale bar: 1 mm. **(A)** Head of the embryo after it was cut away from the remainder of the embryo at forelimb level. **(B)** The rostral part of the head was removed and the remainder of the head tissue positioned so that the 4^th^ ventricle (arrow) was oriented upwards. **(C)** The roof of the 4^th^ ventricle was peeled away, and the hindbrain was exposed by peeling tissue beneath the hindbrain away rostrally and caudally. **(D)** The pial membrane was removed (note that in this example, some cervical spinal cord (SC) tissue has remained attached to the hindbrain). **(E)** Excess midbrain (MB) and spinal cord (SC) tissue has been removed to retain just the hindbrain. **(F)** Culture inserts were coated with laminin and placed into a 12 well tissue culture plate. **(G)** Each hindbrain was placed onto one insert and covered with media. **(H)** Schematic representation of the path taken by migrating FBM neurons (blue) during 24 hr of culture. Click here to view larger image.


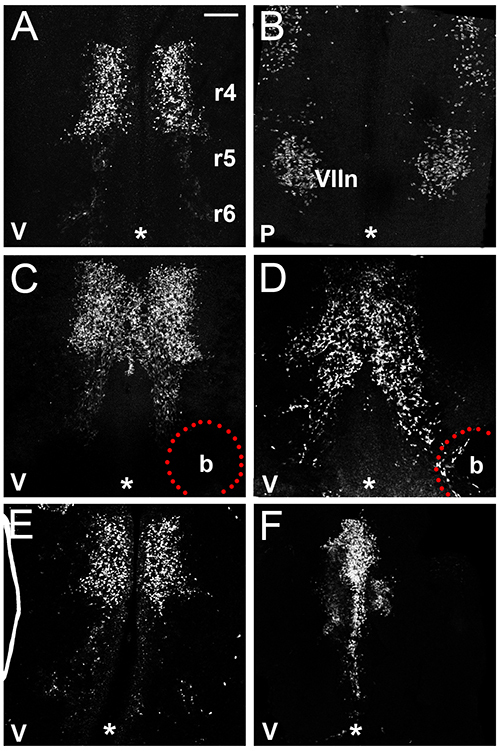
**Figure 3. Mouse hindbrain *ex vivo* culture. (A,B) **An E11.5 hindbrain was cultured for 24 hr and immunofluorescently labeled for ISL1 to illustrate FBM neuron migration in an explant; both ventricular **(A)** and pial **(B)** sides of the hindbrain are shown. **(C,D)** E11.5 littermate hindbrains were cultured in the presence of implanted heparin bead soaked in PBS (**C**) or VEGF165 (**D**); note that FBM neurons migrated towards and onto the VEGF165 bead, and the migrating stream therefore extended further caudally compared to the untreated side of the same hindbrain or the hindbrain containing the control bead. **(E,F)** Examples of unsatisfactory E11.5 hindbrain explants, in which FBMs have not emigrated from r4 **(E)**, or in which the hindbrain tissue folded during culture **(F)**. The midline is indicated with an asterisk in each panel. Scale bar (for all panels): 200 μm. V, ventral; P, pial. Click here to view larger image.

## Discussion

This protocol describes the wholemount culture of E11.5 mouse hindbrains in a transwell system to study the migration of FBM neurons. This protocol allows mouse hindbrain motorneurons to be kept alive and migrating for a period of 24 hr, enabling *ex vivo* manipulation. This method has numerous experimental advantages for investigators seeking to identify the molecular and cellular mechanisms of neuronal migration. Whereas traditional migration assays explant small neural tissue pieces into matrix on culture dishes and enable observation of individual neurons as they respond to exogenous stimuli, a major advantage of the transwell assay is its suitability to manipulate migrating neurons within the host organ environment and therefore a more physiological context. Importantly, substances can be readily applied to the *ex vivo* hindbrain explants to test their effect on neuronal migration, circumventing possible side effects associated with administering these substances to a pregnant mouse. Finally, the *ex vivo* model also allows the testing of substances that do not cross the placental barrier, such as function-blocking antibodies. Due to these advantages, the *ex vivo* hindbrain culture provides an alternative and complementary method to using zebrafish embryos, which can be treated with water soluble small molecules in the aquarium water, or to *in utero* electroporation of embryonic brains, which requires the use of specialized equipment and is more difficult to master than the culture technique described here. Another advantage of the protocol described here is its amenability to implanting beads soaked in recombinant protein or other reagents, therefore allowing the application of a standard embryological method developed to manipulate chick embryos to a mouse model of neuronal migration. In particular, the *ex vivo* culture model may be applied to hindbrains of genetically engineered mice defective in specific molecules implicated in neuronal migration, such as growth or guidance factor receptors, and combined with bead implants to test if responsiveness to ligands is lost. In addition to pharmacological manipulation, the *ex vivo* culture protocol could also be adapted to electroporate expression vectors that could manipulate expression of genes of interest; appropriate methods for electroporation have previously been described^22,23^. This protocol may also be adapted to visualize neuron migration by time-lapse microscopy in hindbrain explants from transgenic mice containing fluorescently labeled FBM neurons, *e.g.* *Isl1-Cre; Rosa26Yfp*^ 21^. Finally, this protocol may also be used to study other types of migrating neurons in the hindbrain, such as those that form the inferior olive, although this would require the use of hindbrains at older embryonic stages and may require culture for up to 48 hr, depending on neuronal viability *ex vivo*.

**Critical steps and troubleshooting**

For the success of this protocol, it is crucial that embryos are collected early on day E11.5, closer to E11.25, when FBM neuron migration has just begun. However, it is not always possible to catch embryos at this developmental stage due to natural mating variability of mice, and accordingly, there may be some variability in the extend of FBM migration between different experiments. Variability in FBM migration may also be observed if the experiment is not completed within the time frame assigned, about 3 hr, as can be seen in **Figure 3E**. Hindbrain tissue from E11.25 mouse embryos is delicate. When dissecting and throughout the explant procedure, it is important to not tear the hindbrain tissue in the areas from r4-r6 where the FBM neurons are located. Due to the delicate nature of the dissection process, and because the speed at which hindbrains are placed into culture influences outcome, the procedure might take a couple of practice runs to master, in particular before precious samples or reagents are used. Finally, it is important that the hindbrain tissue is placed into an open book configuration on the culture insert, because folding of the hindbrain tissue during culture will prevent normal FBM migration (see **Figure 3F**).

## Disclosures

None of the authors have competing interests or conflicting interests.
